# Alterations in brain functional connectivity in patients with mild cognitive impairment: A systematic review and meta‐analysis of functional near‐infrared spectroscopy studies

**DOI:** 10.1002/brb3.3414

**Published:** 2024-04-14

**Authors:** Shuangyan Wang, Weijia Wang, Jinglong Chen, Xiaoqi Yu

**Affiliations:** ^1^ Department of Geriatric Neurology, Guangzhou First People's Hospital The Second Affiliated Hospital of South China University of Technology Guangzhou Guangdong China; ^2^ Department of Library Sun Yat‐sen University Guangzhou Guangdong China

**Keywords:** functional connectivity, functional near‐infrared spectroscopy, mild cognitive impairment, resting state, working memory task

## Abstract

Emerging evidences suggest that cognitive deficits in individuals with mild cognitive impairment (MCI) are associated with disruptions in brain functional connectivity (FC). This systematic review and meta‐analysis aimed to comprehensively evaluate alterations in FC between MCI individuals and healthy control (HC) using functional near‐infrared spectroscopy (fNIRS). Thirteen studies were included in qualitative analysis, with two studies synthesized for quantitative meta‐analysis. Overall, MCI patients exhibited reduced resting‐state FC, predominantly in the prefrontal, parietal, and occipital cortex. Meta‐analysis of two studies revealed a significant reduction in resting‐state FC from the right prefrontal to right occipital cortex (standardized mean difference [SMD] = −.56; *p* < .001), left prefrontal to left occipital cortex (SMD = −.68; *p* < .001), and right prefrontal to left occipital cortex (SMD = −.53; *p* < .001) in MCI patients compared to HC. During naming animal‐walking task, MCI patients exhibited enhanced FC in the prefrontal, motor, and occipital cortex, whereas a decrease in FC was observed in the right prefrontal to left prefrontal cortex during calculating‐walking task. In working memory tasks, MCI predominantly showed increased FC in the medial and left prefrontal cortex. However, a decreased in prefrontal FC and a shifted in distribution from the left to the right prefrontal cortex were noted in MCI patients during a verbal frequency task. In conclusion, fNIRS effectively identified abnormalities in FC between MCI and HC, indicating disrupted FC as potential markers for the early detection of MCI. Future studies should investigate the use of task‐ and region‐specific FC alterations as a sensitive biomarker for MCI.

## Graphical abstract

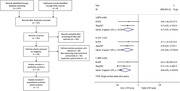

## 1 INTRODUCTION

Mild cognitive impairment (MCI) represents an intermediary phase between typical cognitive aging and more severe neurodegenerative conditions, such as Alzheimer's disease (AD). Marked by a progressive decline in motor, cognitive, and linguistic abilities surpassing age‐related expectations (Nichols et al., 2019), the identification of robust biomarkers for early MCI detection is crucial. Such biomarkers could facilitate timely intervention and effective management, potentially mitigating the progression to more debilitating cognitive disorders like AD (Joe & Ringman, 2019; Livingston et al., 2020).

Cognitive function hinges on intricate interactions among various brain regions, forming functional networks (van den Heuvel & Hulshoff Pol, 2010). Functional connectivity (FC) captures the dynamic synchronization of neural activity across these regions, elucidating their cooperative function (Fox & Raichle, 2007). FC plays a pivotal role in unraveling complex interactions among brain regions, revealing the subtle changes occurring in brain networks before clinical symptoms manifest (Canuet et al., 2012; Kabbara et al., 2018). Moreover, it offers insights into the underlying mechanisms governing both cognitive function and dysfunction. Therefore, the analysis of FC has emerged as a valuable tool for investigating changes in brain network organization associated with MCI (Wang et al., 2013).

Functional near‐infrared spectroscopy (fNIRS), a noninvasive neuroimaging technique, has gained recognition as a promising approach for exploring brain function and connectivity (Ferrari & Quaresima, 2012). By quantifying fluctuations in oxygenated and deoxygenated hemoglobin concentrations in response to neuronal activity, fNIRS provides a unique perspective into the functional dynamics of the brain (Naseer & Hong, 2015; Quaresima & Ferrari, 2019). The portability, cost‐effectiveness, and participant‐friendliness characteristics of fNIRS make it particularly suitable for investigating alterations in brain FC in clinical populations (Scholkmann et al., 2014).

Although previous research studies have explored the FC of individuals with MCI using fNIRS across various cognitive tasks, outcomes have shown variability among studies (Tang & Chan, 2018; Vermeij et al., 2017; Yap et al., 2017; Yeung et al., 2016a, 2016b). Therefore, this systematic review and meta‐analysis aim to thoroughly evaluate the current body of literature regarding alterations in brain FC in MCI patients via fNIRS. Specifically, this review seeks to achieve several objectives: (1) provide a comprehensive overview of FC alterations in MCI populations utilizing fNIRS, (2) identify consistent patterns of FC changes that may underlie cognitive deficits, (3) explore potential correlations between FC changes and cognitive or clinical measures, and (4) assess the potential of fNIRS‐based FC as a valuable biomarker for MCI.

## 2 MATERIALS AND METHODS

The present systematic review and meta‐analysis adhered to the guidelines outlined in the preferred reporting items for systematic reviews and meta‐analysis (PRISMA).

### 2.1 Search strategy

Databases, including PubMed, Web of Science, EMBASE, and Scopus, were systematically searched for human subject studies published in English from database inception to July 12, 2023. The following search terms were employed: “cognitive impairment OR MCI” AND “FC OR brain network OR brain connectivity” AND “near infrared spectroscopy OR fNIRS.” Manual searches were conducted in the reference lists of included studies and relevant review articles to identify additional pertinent studies. Two independent reviewers performed the literature search, and any disparities were resolved through consensus.

### 2.2 Eligibility criteria

The inclusion criteria were as follows: (1) Experiment group included participants with MCI or amnestic MCI (aMCI); (2) control group included age‐matched healthy participants without any type of cognitive impairment; (3) participants in both groups underwent fNIRS assessments to monitor the cerebral hemodynamic response during resting‐state or under various cognitive tasks; and (4) FC was measured utilizing fNIRS signals. The exclusion criteria were as follows: (1) patient with AD; (2) studies lacking FC measurements; and (3) single‐arm investigations, nonhuman experiments, case reports, conferences abstracts, and studies that did not provide adequate FC data. Two independent reviewers assessed the eligibility of potential publications, and disagreements were resolved through consensus.

### 2.3 Data extraction

A standardized data extraction form was employed to collect methodological and outcome variables from each selected study. These variables included author(s), year of publication, study design, sample size, participant characteristics (i.e., age, sex, and pathology), task paradigms, features of fNIRS setting (i.e., cortical regions of interest [ROI] monitored, wavelength, number of channels, distance from transmitter to detector, and sampling frequency), fNIRS data processing features (i.e., filter frequency, type of cerebral hemodynamic measures), and features of FC analysis (i.e., normalization method). Two reviewers independently performed data extraction, and disagreements were resolved through consensus.

### 2.4 Quality assessment

The methodological quality of the included studies was evaluated using the National Institutes of Health's Quality Assessment tool for Observational Cohort and Cross‐sectional Studies (Health, 2014). We tailored this assessment tool by introducing several modifications to align with the unique characteristics of the studies under consideration. The assessment tool comprises 14 criteria questions, with Questions 10, 12, and 13 deemed inapplicable to the included studies due to the absence of blinded methods and follow‐up assessments. Consequently, each study underwent evaluation based on 11 questions. For each question, the study was categorized as “Good (fully met),” “Fair (partially met),” or “Poor (unmet or not mentioned),” based on the level of potential bias.

### 2.5 Statistical analysis

Meta‐analysis was conducted when data from two or more studies were available. Statistical analyses were performed using Stata software version 15.0 (Stata Corporation). Because functional equivalence was not expected to hold across the included studies, and a common effect size could not be assumed, we performed a random‐effects meta‐analysis (Borenstein et al., 2009). Mean ± standard deviations (SDs) of relevant FC outcomes were extracted and pooled using standardized mean difference (SMD) with corresponding 95% confidence interval (CI). Effect size was determined using Hedges' g to account for potential biases in studies with limited sample sizes. Studies were excluded from the meta‐analysis if they meet the following criteria: fewer than two studies examining the same ROI, absence of SD values, absence of control data, or a presentation of control and MCI groups as mixed data. Heterogeneity among the studies was assessed using Cochran's *Q* test and quantified using *I* ^2^ statistic. The degree of heterogeneity, as represented by *I* ^2^, was interpreted as follows: modest (*I* ^2^ ≤ 25%), moderate (25% < *I* ^2^ ≤ 50%), substantial (50% < *I* ^2^ ≤ 75%) or considerable (*I* ^2^ > 75%) (Littell et al., 2008). All analyses employed two‐sided *t*‐tests, with *p*‐values <.05 considered statistically significant.

## 3 RESULTS

### 3.1 Search results

**FIGURE 1:**
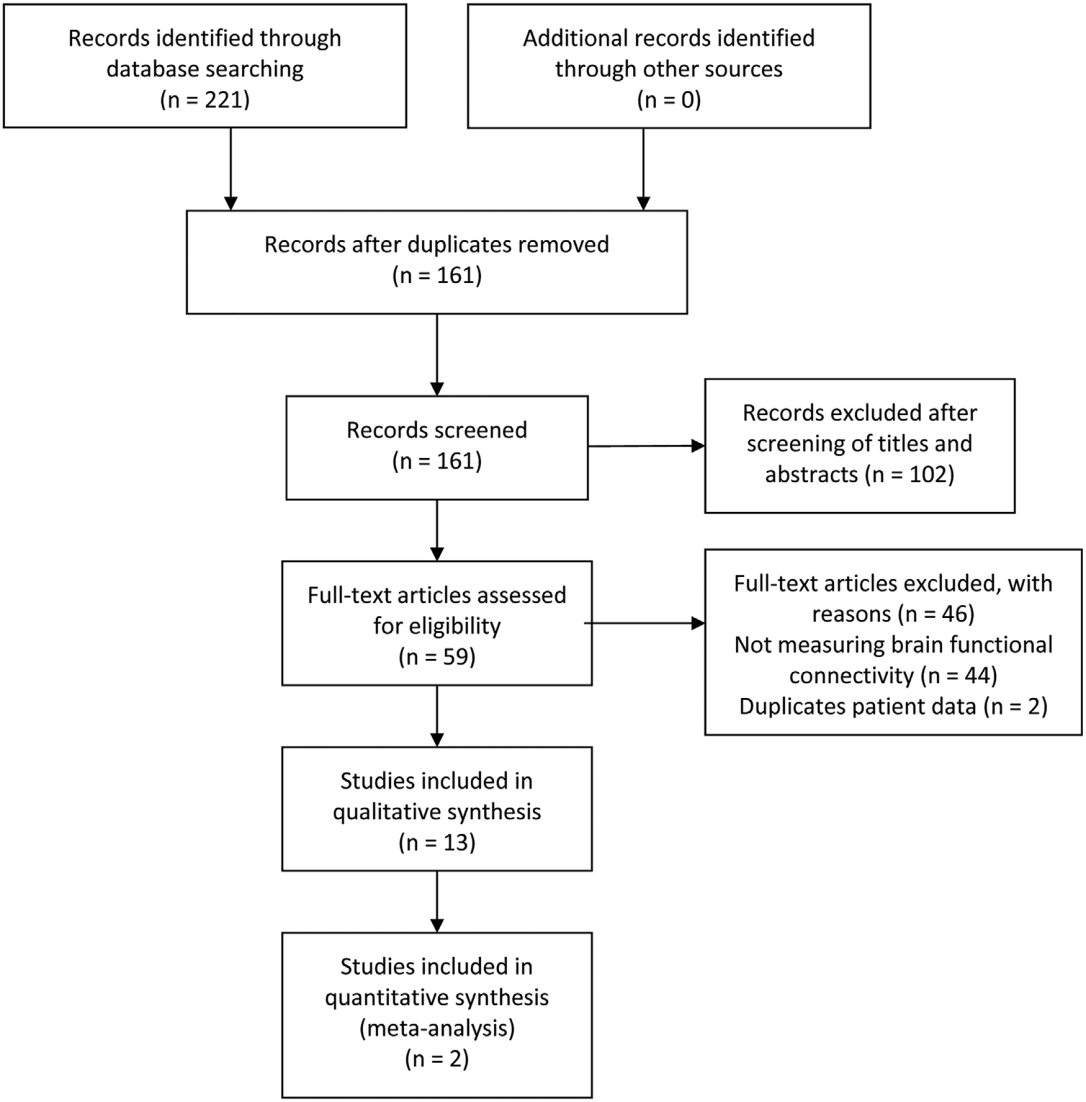
The preferred reporting items for systematic reviews and meta‐analysis (PRISMA) flow diagram presenting the process of study selection.

The initial search yielded a total of 221 references. Following removal of duplicates and screening of titles/abstracts, 59 publications remained eligible for full‐text review. Eventually, 13 studies were included in this systematic review and meta‐analysis. Among them, 13 studies were included in the qualitative synthesis (Bu et al., 2019; Ghafoor et al., 2019; Li et al., 2019; Liu et al., 2021; Nguyen et al., 2019; Niu et al., 2019; Tang & Chan, 2018; Wang et al., 2022; Yang & Hong, 2021; Yoo & Hong, 2019a, 2019b; Yu et al., 2020; Zhang et al., 2022), whereas only 2 studies met the criteria for quantitative synthesis (Bu et al., 2019; Zhang et al., 2022). The PRISMA flow diagram of the literature search and study selection strategy is shown in Figure 1.

### 3.2 Study characteristics

The 13 studies encompassed a total of 304 MCI patients (105 aMCI) and 337 age‐matched healthy control (HC) subjects. Participants’ cognitive status was assessed using standardized screening tools such as the mini‐mental state examination (MMSE) and the Montreal Cognitive Assessment (MoCA). Experimental paradigms exhibited diversity. Resting‐state FC was measured in eight studies, with task duration ranging from 30 s to 15 min. Simple‐walking and dual‐task walking FC was examined in two studies. In five studies, FC was measured during working memory tasks, including “memory‐retrieval” digits verbal span task, oddball task, N‐back task, and the delayed matching to sample (DMTS) task. Three studies have measured FC during a semantic verbal fluency task. Table 1 provides a summary of the basic characteristics of the included studies.

**TABLE 1:** Basic Characteristics of the included studies.

Author	Region	MCI^a^ (N)	Age (years)	Sex (M/F)	MMSE	HC (N)	Age (years)	Sex (M/F)	MMSE	Tasks
Bu et al. (2019)	China	26	69.3 ± 3.6	13/13	26.15 ± 1.67	28	70.2 ± 3.5	14/14	29.43 ± 0.5	RS
Ghafoor et al. (2019)	Korea	12	61.6 ± 6.6	0/12	–	12	55.9 ± 7.7	0/12	–	RS; WM task
Li et al. (2019)	China	16	67.6 ± 8	9/7	26 ± 2.45	16	61.2 ± 10.5	10/6	27.93 ± 1.61	DVST
Liu et al. (2021)	China	23	62.4 ± 5.0	–	27.64 ± 1.76	22	64.4 ± 5.5	–	29.09 ± 1.15	Walking; DT walking
Nguyen et al. (2019)	Korea	42	75.9 ± 3.6	–	–	42	74.3 ± 4.4	–	–	RS; oddball; 1‐back; VFT
Niu et al. (2019)	China	25	71.0 ± 8.1	12/13	23.48 ± 4.81	30	67.6 ± 9.0	11/19	28.23 ± 3.07	RS
Tang and Chan (2018)	Malaysia	12	73.1 ± 8.2	8/4	–	31	72.6 ± 8.5	19/12	–	SVFT
Wang et al. (2022)	China	16	69.7 ± 6.5	4/12	22.93 ± 2.13	38	67.9 ± 7.4	11/27	28.36 ± 1.58	Walking; DT walking
Yang and Hong (2021)	Korea	15	69.3 ± 7.1	1/14	25.13 ± 2.33	9	68.3 ± 4.7	2/7	27.22 ± 1.98	RS
Yoo and Hong (2019a)	Korea	15	69.3 ± 7.1	1/14	25.13 ± 2.33	11	69.1 ± 5.1	3/8	69.09 ± 5.11	RS; SVFT
Yoo and Hong (2019b)	Korea	15	63.2 ± 5.5	10/13	–	23	61.2 ± 6.5	11/12	–	RS; N‐back
Yu et al. (2020)	Korea	23	67.5 ± 4.9	29/35	24.74 ± 1.21	64	67.0 ± 5.2	37/27	29.09 ± 0.73	DMTS
Zhang et al. (2022)	China	64	69.3 ± 3.6	13/13	25.31 ± 2.43	28	70.2 ± 3.5	14/14	27.72 ± 1.65	RS

Abbreviations: DMTS, delayed matching to sample; DT, dual‐task; DVST, digit verbal span task; HC, healthy control; MCI, mild cognitive impairment; M/F, male/female; MMSE, mini‐mental state examination; RS, resting‐state; SVFT, semantic verbal fluency task; VFT, verbal fluency task; WM, working memory.

^a^ Li et al. (2019), Niu et al. (2019), and Zhang et al. (2022) included patients with amnestic mild cognitive impairment.

All fNIRS devices were designed as continuous wave measurement systems and used a clinically safe light wavelength, ranging from 780 to 850 nm. The number of channels used ranged from 14 to 71, with one study applied a four‐channel fNIRS device (Nguyen et al., 2019). The distance between the emitter and the detector primarily ranged around 30 mm in most studies, with flexibility observed in one study (25–30 mm) (Ghafoor et al., 2019), and variations of 32 and 15 mm in two separate studies. Sampling rates ranged from 3.91 to 50 Hz (Niu et al., 2019; Yu et al., 2020).

All studies reported cortical hemodynamic responses using oxygenated hemoglobin (HbO), whereas five studies additionally presenting results for deoxygenated hemoglobin (HbR). The prefrontal cortex emerged as the most frequently monitored brain region, with 54% of the included studies reporting placement solely over the prefrontal cortex. Six studies (46%) reported placement over the prefrontal cortex as well as the occipital, motor, parietal, and temporal cortexes. Data preprocessing encompassed several steps, including the conversion of raw data to relative changes in HbO and HbR concentration, band‐pass filtering, motion artifact correction, and spatial normalization. A detailed overview of the fNIRS device features and FC analysis methods is presented in Table 2.

**TABLE 2:** Characteristics of functional near‐infrared spectroscopy (fNIRS) features of the included studies.

Author	No. of channels	Wavelengths	Emitters and detectors	Sampling rate	Obtain signals	ROI	Inter‐optode distances	FC analysis method
Bu et al. (2019)	14	780, 808, and 850 nm	16 s and 16 detectors	10 Hz	HbO	Prefrontal, motor, and occipital cortex	30 mm	Dynamic bayesian inference
Ghafoor et al. (2019)	20	760 and 850 nm	8 s and 7 detectors	7.81 Hz	HbO	Prefrontal cortex	25–30 mm	*z*‐Scores
Li et al. (2019)	30	760 and 850 nm	16 emitters and 16 detectors	3.91 Hz	HbO	Prefrontal, parietal, and occipital cortex	30 mm	Pearson's correlation coefficients
Liu et al. (2021)	14	780 and 830 nm	52 sources and 54 detectors	10 Hz	HbO	Prefrontal, motor, and occipital cortex	30 mm	Wavelet phase coherence, amplitude‐adjusted Fourier transform
Nguyen et al. (2019)	6	730 and 850 nm	2 emitters and 5 detectors	8 Hz	HbO_,_ HbR, HbT	Prefrontal cortex	Long channel: 30 mm; short channel: 8 mm	*z*‐Scores
Niu et al. (2019)	46	690 and 830 nm	12 emitters and 24 detectors	50 Hz	HbO	Entire cortex	32 mm	Sliding window‐based correlation; FC variability index (*Q*)
Tang and Chan (2018)	52	690 and 830 nm	17 sources and 16 detectors	10 Hz	HbO	Prefrontal cortex	30 mm	Pearson's correlation coefficients
Wang et al. (2022)	43		23 sources and 16 detectors	8.138 Hz	HbO and HbR	Prefrontal, parietal, temporal, and occipital cortex		
Yang and Hong (2021)	48	780 and 850 nm	24 emitters and 32 detectors	8.138 Hz	HbO and HbR	Prefrontal cortex	30 mm	Pearson's correlation coefficients
Yoo and Hong (2019a)	48	780 and 850 nm	24 sources and 32 detectors	8.138 Hz	HbO and HbR	Prefrontal cortex	30 mm	Pearson's correlation coefficients
Yoo and Hong (2019b)	48	780 and 850 nm	24 sources and 32 detectors	8.138 Hz	HbO and HbR	Prefrontal cortex	30 mm	Pearson's correlation coefficients
Yu et al. (2020)	1.5 cm‐channels: 52; 3 cm‐channels: 68	780 and 850 nm	24 sources and 32 detectors	8.138 Hz	HbO	Prefrontal cortex	15 and 30 mm	*z*‐Scores
Zhang et al. (2022)	71	730 and 850 nm	22 sources and 31 detectors	19 Hz	HbO	Prefrontal, parietal, temporal, and occipital cortex		*z*‐Scores

Abbreviations: FC, functional connectivity; HbO, oxygenated hemoglobin; HbR, deoxygenated hemoglobin; HbT, total hemoglobin; ROI, regions of interest.

### 3.3 Quality assessment

The distributions of the quality assessments for each study are presented in Table S1. All of the included studies clearly stated their research question or objectives, as well as explicitly defined their study population. Four studies did not provide specific inclusion and exclusion criteria to all participants. Further, 10 studies did not provide justification for their chosen sample sizes. Five studies did not statistically measure and adjust for confounding variables.

### 3.4 Resting‐state functional connectivity

Resting‐state FCs were evaluated in eight studies, consistently revealing distinct FC patterns capable of discriminating between MCI and HC patients.

In five studies focusing on channel‐based FC within the prefrontal cortex, four observed a significantly reduced FC in MCI patients. Ghafoor et al. revealed weakened FC in both intra‐ and interhemispheric prefrontal regions in MCI patients compared with HC. The mean FC metrics were positively correlated with MoCA‐K scores, suggesting this correlation as a prognostic marker of MCI (Ghafoor et al., 2019). In their graph‐theory analysis, Ghafoor et al. further revealed that MCI patients exhibited lower global efficiency, local network efficiencies, small worldness, nodal efficiencies, clustering coefficients, and degree centrality compared with control group, providing additional evidence of disrupted FC in MCI patients (Ghafoor et al., 2019).

Similarly, Yang and Hong (2021) demonstrated weakened FC in the MCI group compared to the HC group across all measurement durations exceeding 90 s. They proposed a novel method for identifying MCI from the HC using transfer learning methods and found that the minimum time window of 30 s in the resting‐state achieved good accuracy (maximum accuracy: 95.81%) for MCI detection (Yang & Hong, 2021).

Applying different thresholds, Yoo et al. (1) (the high correlations over 0.8 returns 1) and Yoo et al. (2) (the high correlations over 0.7 returns 1) both demonstrated a significantly lower number of correlated channels in MCI individuals compared to HC (Yoo & Hong, 2019a, 2019b). In contrast, Nguyen et al. (2019) revealed significantly higher right and interhemispheric connectivity during resting‐state in MCI individuals compared to HC. This finding might be attributed to the compensatory mechanism observed in MCI (Lenzi et al., 2011), or the variations in time and fNIRS device used. In Nguyen et al., a homemade four‐channel fNIRS was utilized, and a 60‐s resting‐state task was performed, which had fewer channels and a shorter duration than other included studies.

Three studies investigated ROI‐based FC covering the prefrontal, occipital, parietal, temporal, and motor cortexes, consistently revealing disrupted FC in prefrontal cortex‐related brain regions. Bu et al. (2019) demonstrated a significant reduction in FC between bilateral prefrontal and occipital cortexes in the MCI group compared with the HC group. Correlation analysis using Kendall's *τ* rank correlation coefficient revealed strong positive correlations between FC values and cognitive scores (MMSE and MoCA), particularly in connections between bilateral prefrontal regions (Bu et al., 2019).

Zhang et al. (2022) initially showed significantly reduced FC in MCI individuals compared to HC from a whole‐brain perspective, indicating widespread cognitive decline. Subsequently, they found significantly reduced FC in the MCI group among bilateral prefrontal, parietal, occipital, and right temporal cortexes compared with HC. Specifically, long‐range connections from prefrontal to occipital cortex and from prefrontal to parietal cortex. Using linear discriminant analysis for classification, they reported a mean classification accuracy of 66.48% for ROI‐based connections, with the highest accuracy of 71.59% located between the right prefrontal and the left occipital cortexes (Zhang et al., 2022).

Niu et al. (2019) utilized sliding‐window correlation and k‐means clustering analyses, revealing increased dynamic FC variability strength (*Q*) and abnormal occurrence frequency (*F*) in aMCI patients compared to HC in specific brain connectivity states, concentrated in regions including the prefrontal and parietal cortexes. The *Q* values were further found to be negatively related to MMSE scores in the intra‐hemispheric long connections from aMCI. Using the *Q* value as a measurement, the classification performance exhibited an accuracy of 82.5% in differentiating aMCI from HC, providing a methodological framework for applying dynamic FC analysis to aMCI patients (Niu et al., 2019).

**FIGURE 2:**
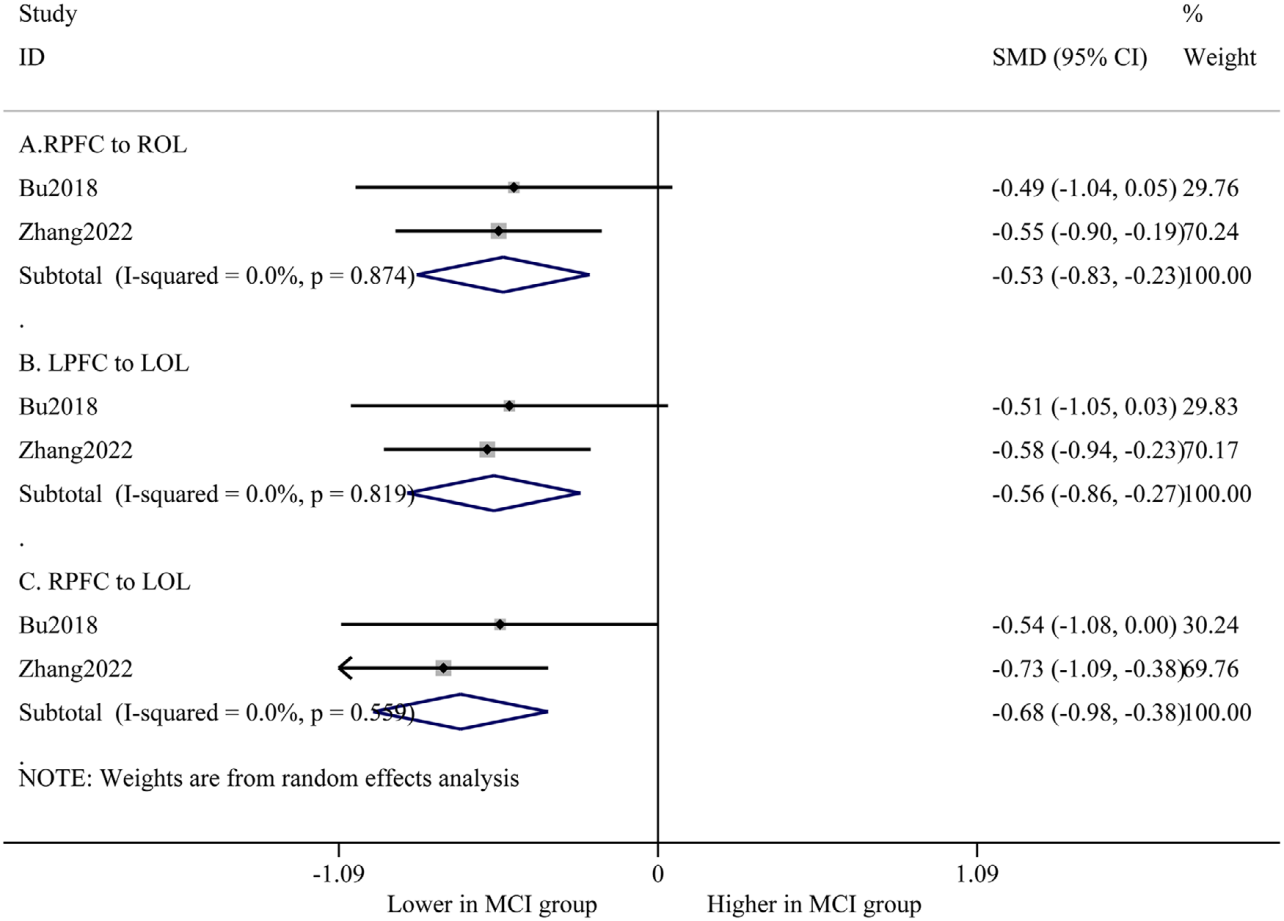
Forest plot of functional near‐infrared spectroscopy (fNIRS)‐based function connectivity between mild cognitive impairment and healthy controls: (A) function connectivity from left prefrontal cortex to left occipital cortex (LPFC to LOL); (B) function connectivity from right prefrontal cortex to left occipital cortex (RPFC to LOL); and (C) function connectivity from right prefrontal cortex to right occipital cortex (RPFC to ROL).

Although utilizing different quantified methods, two studies (Bu et al., 2019; Zhang et al., 2022) assessed resting‐state FC at the same ROI. Meta‐analysis results showed that the FC of the right prefrontal to the right occipital cortex (SMD = −.56; 95% CI, −.86 to −.27; *p* < .001; *I* ^2^ = 0%), the left prefrontal to the left occipital cortex (SMD = −.68; 95% CI, −.98 to −.38; *p* < .001; *I* ^2^ = 0%), and the right prefrontal to the left occipital cortex (SMD = −.53; 95% CI, −.83 to −.23; *p* < .001; *I* ^2^ = 0%) were significantly lower in MCI patients compared to health controls (Figure 2).

### 3.5 Ambulatory task functional connectivity

Two studies, conducted by Liu et al. (2021) and Wang et al. (2022), have measured FC during both single‐ and dual‐task walking. Both studies found no significant differences in FC during single‐task walking between the MCI and HC groups. However, disrupted FC was observed in MCI patients compared with HC during dual‐task walking, suggesting that combining high‐sensitivity subtasks may be helpful for diagnosing and identifying MCI patients.

Liu et al. discovered that the FC of the left prefrontal to the right occipital cortex was significantly higher in the MCI group than that in the HC group during counting backward‐walking task. Additionally, the FC of the left prefrontal to the left motor cortexes was significantly higher in the MCI group than that in the HC group in naming animals‐walking task (Liu et al., 2021). They further demonstrated that the significantly different FC between groups were negatively correlated with relative symmetry index (IDps), suggesting that gait parameters might reflect the abnormal changes in FC in MCI individuals (Liu et al., 2021). Wang et al. (2022) revealed that the FC of MCI patients was generally weakened compared to that of the HC during calculating‐walking task, with the brain regions showing decreased FC mainly associated with the left prefrontal cortex and the right prefrontal cortex.

### 3.6 Working memory task functional connectivity

Four studies have investigated FC during a working memory task, with the majority demonstrating increased FC in the MCI group compared to the HC group.

Three studies focused on channel‐based FC within the prefrontal cortex. Nguyen et al. (2019) utilized a four‐channel fNIRS to record FC and reported similar interhemispheric connectivity between MCI and HC groups during oddball task and 1‐back task. Yoo et al. (2) also found similar connectivity between MCI and HC groups during N‐back task; however, in some channels, such as the medial prefrontal cortex, FC was more correlated in the MCI patients than in the HC (Yoo & Hong, 2019b). Yu et al. (2020) performed a DMTS task and reported greater number and strength of the prefrontal FC in the MCI group compared with HC group. In particular, greater inter‐ and left‐hemispheric FC was observed in the MCI group, some of which were significantly different between groups during retrieval (Yu et al., 2020).

Li et al. (2020) measured whole‐brain FC during a “memory‐retrieval” digits verbal span task, demonstrating stronger FC in aMCI patients between multiple ROI pairs compared to HC. Graph theory analysis indicated higher global efficiency and clustering coefficient in aMCI patients compared to HC, suggesting higher integration and higher segregation in the brain networks of aMCI patients. Notably, aMCI patients exhibited significantly higher clustering coefficient in regions including the left middle frontal, left precentral, bilateral superior temporal, and superior parietal areas. Result of correlation analysis indicated that all global network measures (mean correlation coefficient, global efficiency, and average clustering coefficient) and regional measures (clustering coefficient and centrality) of most identified ROIs were significantly correlated with MMSE and MoCA score (Li et al., 2020).

### 3.7 Verbal fluency task functional connectivity

Three studies have measured FC within the prefrontal cortex during semantic verbal fluency tasks, revealing variable FC patterns with both decreased and increased connectivities in different brain regions.

Nguyen et al. (2019) demonstrated significantly lower left and interhemispheric connectivity in MCI patients compared to HC. Tang et al. found a general decline in FC from HC to MCI patients, along with a significant decreased in the clustering coefficient and small worldness of the prefrontal cortex network from HC to MCI. This suggests a loss of regularity and a higher dispersion in brain network of MCI patients (Tang & Chan, 2018). Tang et al. further revealed that connectivity in HC was more concentrated in the left and middle prefrontal cortex than the right prefrontal cortex, whereas the concentrated distribution shifted from left to right prefrontal cortex in MCI group. Yoo et al. (1) a significantly lower number of correlated channels in the MCI group than in the HC group (Yoo & Hong, 2019a). They further showed that activation in Broca's area was significantly lower in MCI group compared with the HC group, whereas the activation in the Brodmann area on the right hemisphere was higher in the MCI patients (Yoo & Hong, 2019a).

## 4 DISCUSSION

The incorporation of FC analysis with fNIRS has expanded the scope of comprehensive MCI management. This noninvasive approach, offering real‐time feedback, facilitates the tracking of MCI progression and the exploration of intricate mechanisms underlying MCI across various cognitive tasks (Bonilauri et al., 2020; Pinti et al., 2020). Despite limitations such as small sample sizes and heterogeneity, our systematic review and meta‐analysis consistently demonstrated distinct abnormalities in brain FC between MCI and HC groups. Results from our study revealed different FC alterations across different cognitive tasks and brain regions, indicating task‐ and region‐specific FC distributions. Therefore, recognizing consistent FC patterns for specific tasks and brain regions is crucial for facilitating the early detection and management of MCI.

In the context of resting‐state, individuals with MCI consistently exhibited reduced FC in the bilateral prefrontal cortexes as well as in the prefrontal‐related long‐distance connections with the occipital, parietal, and temporal cortexes. This reduction in resting‐state FC may be attributed to hypoperfusion and hypometabolic patterns in MCI patients, leading to diminished tissue oxygenation levels and weaker blood flow turbulence. Clinically, these alterations manifest as compromised brain synchronization (Brier et al., 2012; Wang et al., 2015).

Dual‐task paradigms have gain increased popularity in brain studies, providing a gold standard for evaluating the “central executive system” (Bishnoi et al., 2021). Studies investigating FC during dual‐task walking have shown mixed findings, revealing both increased and reduced prefrontal‐related connectivity (Liu et al., 2021; Wang et al., 2022). These inconsistencies might be attributed to task differences. Although naming animals‐walking task focuses on verbal fluency and relies on semantic memory, the calculating‐walking task examines working memory and attention. It was observed that MCI individuals were linked to stronger FC between the prefrontal, occipital, and motor cortexes during naming animals‐walking task (Liu et al., 2021). The prefrontal cortex, widely recognized as a key cognitive region, plays a vital role in memory, judgment, analysis, thinking, and operation, (Schättin et al., 2016). The motor cortex primarily coordinates sensory and motor functions (Bugnariu & Fung, 2007), whereas the occipital cortex is involved in visual processing, modulated by planning, executing, and imagining movements (Astafiev et al., 2004; Morgan et al., 2011). This finding indicates that increased visual information processing and sensory coordination are required to regulate the performance of naming animals‐walking task in individuals with MCI. However, weakened bilateral prefrontal cortex FC was found in MCI patients during calculating‐walking task. This suggests that, when confronted with more challenging and sensitive subtasks, the healthy population can maintain the capability to overcome the interference of subtasks on gait. Conversely, individuals with MCI exhibit a relatively inadequate ability to overcome more pronounced subtask interference (Bruce‐Keller et al., 2012).

The results from fNIRS studies investigating FC alterations in MCI patients during working memory tasks exhibit both similarities and differences. Some studies demonstrated similar intra‐ and interhemispheric prefrontal FC between MCI and HC groups during working memory tasks (Nguyen et al., 2019; Yoo & Hong, 2019b). Conversely, other studies revealed stronger FC in MCI patients compared with HC (Li et al., 2020; Yu et al., 2020), particularly involving the medial and left hemispheric prefrontal areas. This strengthened prefrontal FC may be attributed to compensatory mechanisms triggered by hippocampal degeneration. The hippocampus, pivotal for memory function (Eichenbaum, 2004), undergoes volume decrease and FC alterations as cognitive function declines (Farràs‐Permanyer et al., 2015). Our finding aligns with previous evidence demonstrating enhanced prefrontal FC as a compensatory response to the decline in FC between the hippocampus and prefrontal cortex (Agosta et al., 2010; Clément et al., 2013; Kircher et al., 2007; Lenzi et al., 2011; Sperling, 2007).

In studies examining fNIRS‐based FC during a semantic verbal fluency task, consistently lower prefrontal cortex FC was detected in the MCI group compared with the HC group, predominantly involving left‐ and interhemispheric connections. Additionally, Yoo et al. (2) and Tang et al. consistently found a more concentrated FC in the left prefrontal cortex in HC, whereas the distribution of FC shifted from left to the right hemisphere in individuals with MCI (Tang & Chan, 2018; Yoo & Hong, 2019a). This shift between hemispheres may be attributed to the concept of neuroplasticity, wherein MCI subjects exhibited hyperactivation in the right prefrontal cortex as a compensatory mechanism to maintain cognitive function under cognitive impairment.

The MMSE and MoCA are widely used neuropsychological tools for assessing cognitive function in MCI patients. To assess the reliability and clinical values of fNIRS‐based FC, correlation analyses between FC metrics and clinical scores were conducted across various studies (Bu et al., 2019; Ghafoor et al., 2019; Li et al., 2020; Niu et al., 2019). Notably, these results consistently demonstrated a significant association of FC metrics with MMSE and MoCA scores during both resting‐state and working memory tasks, underscoring the clinical value of fNIRS‐based FC as a biomarker in the detection of MCI. Furthermore, several studies have conducted classification analysis to further validate the use of FC metrics as biomarkers and found that resting‐state FC had an accuracy of 73.86%–95.81% in identifying MCI from HC (Niu et al., 2019; Yang & Hong, 2021; Zhang et al., 2022). Nevertheless, results of correlation analysis also suggested that the significant correlation between FC and cognitive scores might be task‐ and region‐specific (Li et al., 2020). Thus, future more comprehensive studies with larger sample size are warranted to verify the current findings.

Several limitations to the present study should be acknowledged. Although 13 studies contributed to the descriptive analysis, the quantitative meta‐analysis incorporated only 2 studies examining resting‐state FC, thereby limiting the statistical power of our study. Additionally, variations in FC metrics reporting thresholds across different datasets underscore the necessity for standardizing binarization methods. It is worth noting that all included studies originated from scholars in from South Korea, China, and Malaysia, potentially introducing reporting bias to our results.

## 5 CONCLUSION

Results of the present study demonstrated clear disruptions in fNIRS‐based FC patterns in individuals with MCI compared with HC across both resting‐state and various cognitive tasks. Nonetheless, it is crucial to acknowledge that these FC abnormalities may be task‐dependent and region‐specific. During resting‐state, MCI patients consistently demonstrated a reduction in FC, primarily involving the bilateral prefrontal, occipital, and parietal cortexes. In dual‐task walking, enhanced FCs were predominantly found in the prefrontal, occipital, and motor cortexes of MCI patients. During working memory task, stronger FC was found in the MCI patients, particularly involving the medial and left hemispheric prefrontal areas. In verbal frequency task, MCI consistently exhibited lower FC in the left‐ and interhemispheric prefrontal cortex, along with a more concentrated FC in the right prefrontal cortex as compared with HC. Further research studies are warranted to establish standardized protocols for fNIRS‐based FC assessments, validate findings across larger and more diverse populations, and assess the specificity and sensitivity of fNIRS‐based FC alterations as potential biomarkers for MCI.

## AUTHOR CONTRIBUTIONS

**Shuangyan Wang**: Conceptualization; project administration; visualization; writing—original draft; writing—review and editing; funding acquisition; supervision. **Weijia Wang**: Data curation; formal analysis; investigation; writing—review and editing. **Jinglong Chen**: Data curation; investigation; formal analysis; writing—review and editing. **Xiaoqi Yu**: Data curation; formal analysis; investigation; writing—review and editing.

## CONFLICT OF INTEREST STATEMENT

The authors have no potential conflicts of interest to disclose.

### PEER REVIEW

The peer review history for this article is available at https://publons.com/publon/10.1002/brb3.3414.

## Supporting information

Table S1 Quality assessment results.

## Data Availability

All data generated or analyzed during this study are included in this published article.
