# Supplementary material for: Alterations in brain functional connectivity in patients with mild cognitive impairment: A systematic review and meta‐analysis of functional near‐infrared spectroscopy studies
Source: Brain Behav. 2024 Apr 14;14(4):e3414. doi: 10.1002/brb3.3414 (PMC11016629; doi:10.1002/brb3.3414)
Supplement: Supplementary file 1 — Table S1 Quality assessment results. [file BRB3-14-e3414-s001.docx]

Supplementary Table 1: Quality assessment results.

National Institutes of Health’s (NIH) Quality Assessment tool for Observational cohort and Cross-sectional studies

| Author | 1 | 2 | 3 | 4 | 5 | 6 | 7 | 8 | 9 | 10 | 11 | 12 | 13 | 14 |
| --- | --- | --- | --- | --- | --- | --- | --- | --- | --- | --- | --- | --- | --- | --- |
| Bu2018 | Good | Good | Fair | Fair | Good | Good | Good | Good | Good | / | Good | / | / | Good |
| Ghafoor2019 | Good | Good | Good | Good | Good | Good | Good | Fair | Good | / | Good | / | / | Good |
| Li2019 | Good | Good | Good | Fair | Poor | Good | Good | Good | Good | / | Good | / | / | Good |
| Liu2021 | Good | Good | Good | Good | Poor | Good | Good | Good | Good | / | Good | / | / | Good |
| Nguyen2019 | Good | Good | Good | Good | Poor | Good | Good | Good | Good | / | Good | / | / | Good |
| Niu2019 | Good | Good | Fair | Good | Poor | Good | Good | Good | Good | / | Good | / | / | Good |
| Tang2018 | Good | Good | Fair | Good | Poor | Good | Good | Fair | Good | / | Good | / | / | Fair |
| Wang2022 | Good | Good | Poor | Good | Good | Good | Good | Good | Good | / | Good | / | / | Fair |
| Yang2021 | Good | Good | Good | Good | Poor | Good | Good | Good | Good | / | Good | / | / | Good |
| Yoo2019(1) | Good | Good | Good | Fair | Poor | Good | Good | Poor | Good | / | Good | / | / | Poor |
| Yoo2019(2) | Good | Good | Good | Fair | Poor | Good | Good | Poor | Good | / | Good | / | / | Poor |
| Yu2020 | Good | Good | Good | Good | Poor | Good | Good | Good | Good | / | Good | / | / | Good |
| Zhang2022 | Good | Good | Good | Good | Poor | Good | Good | Good | Good | / | Good | / | / | Fair |

**For each question, study was rated as “Good (completely met)”, “Fair (partially met)”, or “Poor (unmet or not mention)” based on the level of risk of bias.
Since blinded method and follow-up assessment were not applicable of the included studies, Question 10, 12, and 13 were not evaluated.
Item1-** Was the research question or objective of the study clearly stated?
**Item2-** Was the study population clearly specified and defined?
**Item3-** Was the participation rate of eligible persons at least 50%?
**Item4-** Were inclusion and exclusion criteria for being in the study prespecified and uniformly applied to all participants?
**Item5-** Was sample size justification, power description or variance and effect estimates provided?
**Item6-** For analysis of paper, were the exposure(s) of interest measured prior to the outcome(s) being measured?
**Item7-** Was the timeframe sufficient so that one could reasonably expect to see an association between exposure and outcome if it existed?
**Item8-** For exposures that can vary in amount or level, did the study examine different levels of the exposure as related to the outcome (e.g., categories of exposure, or exposure measured as continuous variable)?
**Item9-** Were the exposure measures (independent variables) clearly defined, valid, reliable, and implemented consistently across all study participants?
**Item10-** Was the exposure(s) assessed more than once over time?
**Item 11**- Were the outcome measures (dependent variables) clearly defined, valid, reliable, and implemented consistently across all study participants?

**Item 12**- Were the outcome assessors blinded to the exposure status of participants?

**Item13-** Was loss to follow-up after baseline 20% or less? **Item 14**- Were key potential confounding variables measured and adjusted statistically for their impact on the relationship between exposure(s) and outcome(s)?
